# Measles in London

**Published:** 1905-02-04

**Authors:** 


### The Hospital.

A Journal of

The Medical Sciences and Hospital Administration.

Vol. XXXVII.—No. 958. FEBRUARY 4, 1905.

### MEASLES IN LONDON.

It is shown by recently-published reports that, during the year 1903, as in many previous years, the prevalence and the fatality of measles constituted a marked feature of the general sanitary or insanitary state of the metropolis, and one that manifestly calls for the closest attention on the part of those who are in any way responsible for the care of the public health. The actual mortality produced by the disease in the county of London amounted to 2,048 at all ages; and of these deaths no less than 1,968 were in children under five years of age. Of the remaining 80, 70 were under 10, three under 15, and the remaining seven were over 15. The mortality, heavy as it was, compared favourably with that of the preceding year, in which the deaths were 2,360; but if we glance back over a longer period, we find only fluctuation, with no resemblance to any maintained or steady decrease. The death-rate of the years from 1850 to 1890 ranged from 0·51 to 0·64 per thousand persons living; that of 1896 reached 0·82 per thousand, while that of 1902 returned to 0·51, and that of 1903 fell to 0·44, or about the level of the three years 1899–1901, a diminution possibly due in part to the exhaustion of the more susceptible part of the infant population. If we compare the fatality of measles with that of other infantile diseases, we find that the deaths occasioned by it in 1903, a year of somewhat low prevalence, exceeded those occasioned by whooping-cough by 431, those occasioned by diphtheria by 1,308, those occasioned by scarlet fever by 1,687, and those occasioned by small-pox by 2,035. Moreover, for the decade 1893–1902 the Metropolitan death-rate from measles exceeded that of all other districts except Manchester, West Ham, Salford, and Newcastle; and in 1903 it overtook and surpassed that of Newcastle. Of the extreme infectivity of the disease there can be no question on the part of any experienced practitioner, and it may safely be presumed that, especially in the more inclement months of the year, the case mortality bears a large proportion to the prevalence. Yet, notwithstanding the grave character of the facts to be dealt with, it was not until 1902 that the London County Council extended to measles any part of the provisions of the London Public Health Act of 1891, and these provisions did not become operative until April 1st, 1903. Even now the disease is not "notifiable," and the sanitary authorities are therefore left without any information concerning either its actual prevalence or the percentages of its case mortality.

The notification of occurring cases would, it may be freely admitted, be less serviceable in the case of measles than in many other diseases, for the simple reason that it is freely infectious for a period of at least three days before the appearance of the eruption allows it to be identified with certainty, so that in many cases the stable door could not be shut until after the steed had been stolen. This consideration, however, seems to call for increased rather than for diminished precaution; and it is obvious that, in the case of a school, notification might be extremely effective in arresting a diffusion of contagion, which would otherwise be indefinite and prolonged. The principal provisions of the Act under which the disease has now been brought are all highly important upon paper, and are calculated to be highly useful in dealing with many other diseases, but we doubt whether they are likely to be of much effect as regards the occurrence of measles among the children of the metropolitan poor. They prohibit the exposure in a public place of a person suffering from the disease, or the transmission, without proper precautions, of infected articles. There are other provisions of a like kind which it would be tedious to specify, but which are not likely to be brought into practical operation until notification can be required. There is among the working classes of London a very general want of recognition of the danger attendant upon measles, and, even if they believe in its infectivity, they are very prone also to regard it as one of the inevitable ills of childhood which must be endured sooner or later and against which it is useless to contend. There are hundreds of "mean streets" in which young children in the early and infective stage of measles would be permitted to play about quite freely, and in which measly infants would be carried by their mothers when bent upon either necessary or unnecessary errands, either upon making purchases at the "shop," or upon gossipping with the neighbours. Neither the shop nor the neighbour would be in the least likely to remind any such offender of the Public Health Act, still less to make any endeavour to enforce it against her. Notification, even if it were nothing else, would at least be an educational agency, and might in time be expected to develop a recognition of the really formidable character of the disease, and thus, indirectly, to secure greater care of the sufferers than it is probable that they now commonly receive. In his report for 1902 Sir Shirley Murphy dwelt upon this aspect of the matter, and pointed out that, in view of the extent to which measles mortality is directly attributable to pulmonary complications it might be practicable to diminish it by the provision of a sick nurse or by the removal of the sufferer to a children's hospital. These suggestions seem to partake somewhat too much of the character of counsels of perfection. Sick nurses are admirable people, but they cannot alter facts; and the physical conditions of the dwelling of a poor family are seldom such as to admit of a combination of warmth with ventilation. As for the hospitals, their resources would be still more severely taxed than at present, if any demand were made upon them for the admission of even a small proportion of the annually-recurring measles of the metropolis. Without notification we shall never know the percentage of mortality; but if we assume it to be only 10, the prevalence of 1903 would have furnished over 20,000 cases. Moreover, in many instances increased hospital facilities would be little more than direct inducements to parental neglect.

